# Dermatosis in dialytic chronic kidney failure

**DOI:** 10.1590/2175-8239-JBN-2021-0227

**Published:** 2022-02-28

**Authors:** Marina Luiza Dalla Costa Favero, Danielle Carvalho Quintella, Nurimar Conceição Fernandes

**Affiliations:** 1 Universidade Federal do Rio de Janeiro, Hospital Universitário Clementino Fraga Filho, Serviço de Dermatologia, Rio de Janeiro, RJ, Brasil.; 2 Universidade Federal do Rio de Janeiro, Hospital Universitário Clementino Fraga Filho, Departamento de Patologia, Rio de Janeiro, RJ, Brasil.

## Clinical Case

**Figure 1:**
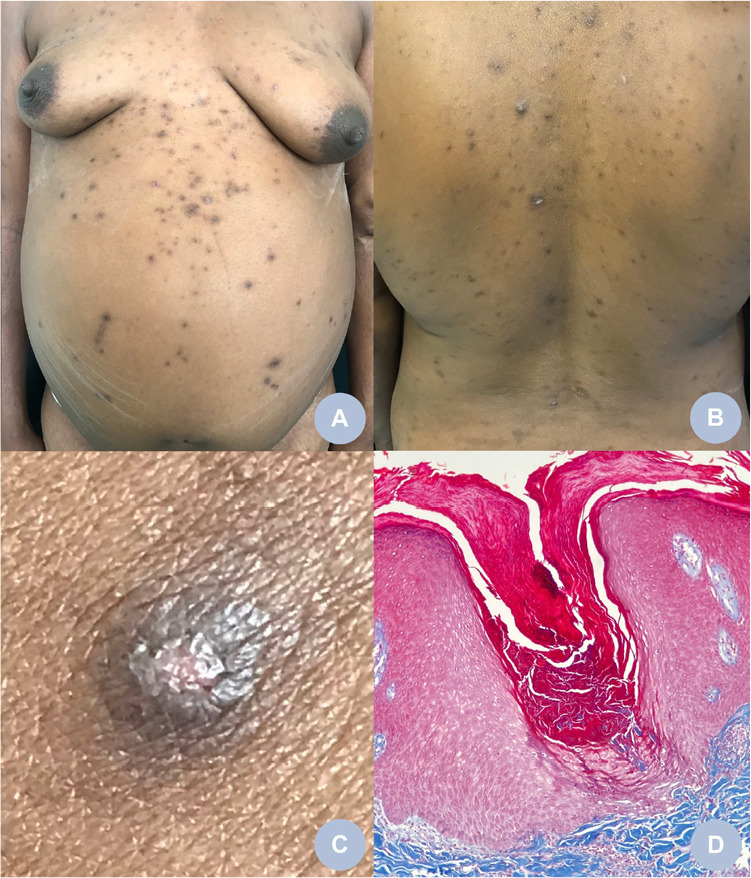
Brownish hyperchromic macules and papules, some keratotic papules with central umbilication on the trunk (A and B). Brownish hyperchromic papules with central umbilication and mild keratosis (C). Masson's trichrome staining shows an area of epidermis invagination and transepidermal elimination of collagen (Masson's trichrome, 100X) (D).

A woman, 40 years old, brown colored skin, presented with papular lesions, generalized pruritus and intense for 2 years. Pathological history revealed a previous diagnosis of systemic arterial hypertension (SAH), type II insulin dependent DM, chronic kidney disease (CKD) stage 5 (anuric) and in hemodialysis for 3 years. She had a past history of ischemic stroke 5 years ago and suffered from dysarthria. On clinical examination, congestive hepatomegaly and ascites were noted. The patient reported irregular use of medications and smoking a pack a day for 20 years. Dermatological examination revealed multiple brownish hyperchromic papules, umbilical, keratotic, some with a cratered center and darkened plugs, pruritic, more pronounced around on the trunk and extensor sides of the upper and lower limbs. Intense cutaneous xerosis and linear excoriations adjacent to the cutaneous lesions were also observed (Figure 1 A, B, C, D)^1,2,3^.

Informed consent was obtained for the publication of this case.

Question 1. Dialytic chronic renal failure, diabetes mellitus and pruritus combined with umbilicated keratotic papules are diagnostic clues for the following dermatosis:

- Lichen simplex shronicus
- Keratosis pillar
- Prurigo nodularis
- Acquired perforating dermatosis

The acquired perforating dermatosis (APD) describes perforating dermatoses that affect adults with diabetes mellitus, chronic renal failure, and rarely other systemic diseases, regardless of the dermal material eliminated^1^. In patients with CKD, APD usually appears after starting dialysis, as in the case reported, and when submitted to kidney transplantation, it tends to be resolved^3^. A histopathological exam with staining by Masson’s trichrome demonstrated transepidermal elimination of collagen (Figure 1 D) and orcein staining revealed preservation of elastic fibers. The diagnosis of acquired perforating dermatosis was based on clinical, histopathological, and onset findings at 38 years of age^4,5^.
