# Erratum: Dermatosis in dialytic chronic kidney failure

**DOI:** 10.1590/2175-8239-JBN-2021-0227er_en

**Published:** 2022-07-25

In the article “**Dermatosis in dialytic chronic kidney failure**”^1^, with DOI code number https://doi.org/10.1590/2175-8239-JBN-2021-0227, published in the Brazilian Journal of Nephrology, ahead of print, 2022:

Where it was written:

Lichen simplex **shronicus**

Should read:

Lichen simplex **chronicus**

